# Evaluating Antioxidant Performance, Biosafety, and Antimicrobial Efficacy of *Houttuynia cordata* Extract and Microwave-Assisted Synthesis of Biogenic Silver Nano-Antibiotics

**DOI:** 10.3390/antiox13010032

**Published:** 2023-12-22

**Authors:** Kavya Moorthy, Kai-Chih Chang, Hsiao-Chi Huang, Wen-Jui Wu, Cheng-Kang Chiang

**Affiliations:** 1 Department of Chemistry, National Dong Hwa University, Shoufeng 97401, Taiwan; 810712202@gms.ndhu.edu.tw; 2 Department of Laboratory Medicine and Biotechnology, Tzu Chi University, Hualien 97004, Taiwan; kaichih@gms.tcu.edu.tw (K.-C.C.); 109323108@gms.tcu.edu.tw (H.-C.H.); w200811@gms.tcu.edu.tw (W.-J.W.); 3 Department of Laboratory Medicine, Buddhist Tzu Chi General Hospital, Hualien 97004, Taiwan

**Keywords:** *Houttuynia cordata* extract, microwave-assisted synthesis, antioxidant activity, bactericidal, biosafety

## Abstract

From the traditional Chinese medicine point of view, although *Houttuynia cordata* extract (HCE) possesses an incredible amount of phytonutrients and exhibits antioxidant activities, excessive doses of HCE can cause danger to organisms and lead to death. In this study, we first examine HCE’s overall phenolic and flavonoid content, antioxidant efficacy, and antibacterial activity. Results show that HCE is suitable as a bio-reducing agent for the microwave-assisted synthesis of silver nanoparticles (HCE-AgNPs) with enhanced antioxidant and antimicrobial performance. Under an optimized microwave condition (i.e., 100 °C for 10 min), the HCE-stabilized AgNPs were confirmed with a UV-visible peak at 430 nm and 19.7 ± 4.2 nm in size. Physicochemical properties of HCE-AgNPs were extensively characterized by zeta-potential, FT-IR, XRD, and XPS measurements. Compared to the HC extract counterpart, HCE-AgNPs display superior antioxidant activity, higher DPPH scavenging efficiency, and enhanced broad-spectrum bactericidal activity to inhibit the growth of all tested bacterial strains at doses of 2 μg/mL. Biosafety evaluation indicated that HCE-AgNPs are noncytotoxic on human red blood cells. These data show that the microwave synthesis of AgNPs exhibits a great antioxidant ability, superior antibacterial activity, and a trivial hemolytic effect, providing another bactericidal therapy strategy to address the increasing healthcare-associated infections.

## 1. Introduction

Pharmaceuticals derived from plants are extraordinarily abundant and provide superior benefits over synthetic substances in terms of variety, volume of production, and safety [1]. Important pharmaceuticals that are widely used in modern medicine include vinca alkaloids, artemisinin, chloroquine, and morphine. The hunt for novel bioactive compounds derived from plants is never-ending and remains a crucial source of potent medicinal compounds [2]. One such plant, *Houttuynia cordata*, a perennial and herbaceous plant native to East Asian countries and a member of the Family “Saururaceae”, received particular focus owing to its possible antiviral properties, particularly in light of the recent COVID-19 pandemic [1,3]. Several studies have documented its pharmacological activity against various conditions, including cancer [4], viruses [5], obesity, hyperglycemia [6], and inflammation [7,8]. Flavonoids, phenolic acids, volatile/essential oils, and alkaloids—some of whose therapeutic potential is still unrealized—are among the numerous phytoconstituents isolated from this plant [9]. Moreover, the presence of quercitrin in both the leaves and flower spikes enhances the resilience of blood vessels and exhibits diuretic characteristics. In accordance with traditional Chinese medicine, *Houttuynia cordata* possesses the ability to alleviate heat and purify the body. It also has cough relief, analgesic properties, and anti-inflammatory properties [4,8,10].

One of the most significant challenges confronting public health in the post-antibiotic era is the rise of drug-resistant microbes brought on by inappropriate consumption of antibiotics [11]. The quick emergence of antibiotic resistance makes it essential to monitor resistant strains, use antibiotics appropriately, and look for new antibacterial agents. Biogenic nanomaterial synthesis utilizing diverse plants and microorganisms is a promising method for creating new antibacterial nanomaterials [12]. The use of green synthesis to produce biogenic NPs has several advantages, including the production of stable NPs with various sizes, shapes, compositions, and physicochemical properties, providing an additional active surface area for interaction in the biological environment, the absence of hazardous byproducts, and cost-effectiveness [13,14].

Due to their extensive antimicrobial capabilities and high antioxidant efficiency against various bacteria, silver nanoparticles (AgNPs) are the most commonly employed nano-antibiotics among the different nanoparticles operating in bactericidal therapies [15]. Among the various metals, silver has garnered interest due to its low production cost and distinctive physical and chemical characteristics, while gold is expensive, and copper has poor stability [16]. Due to their larger surface areas and smaller sizes, Ag nanomaterials have been shown to have antidiabetic, antibacterial, and anticancer properties and help promote bone and wound healing while boosting vaccine immunogenicity [14,16,17].

Compared to traditional heating methods, microwave heating has several advantages, as it increases reaction kinetics and initial rapid heating, boosting reaction rates and producing higher-yielding products [18]. In the regime of nanoparticle synthesis, microwave heating plays a vital role because the controlled high temperature generated enhances the nucleation process, which is the initial step in the formation of nanoparticles [18,19,20]. Higher degrees of crystallinity in nanoparticles and more control over the shape and morphology of the resulting nanostructures have already been reported as benefits of microwave heating over conventional heating [18,19].

In this work, we report on a green, microwave-assisted, low-cost, and environmentally safe method for synthesizing silver nanoparticles using *Houttuynia cordata* aqueous leaf extract. This study is focused on enhancing the antioxidant and antibacterial activity of biogenic AgNPs. Nevertheless, various nanoparticles, including gold nanoparticles (AuNPs) and copper oxide nanoparticles (CuO NPs), have previously been produced using extracts from the *Houttuynia cordata* plant [21,22,23]. Sreekanth et al. employed *Houttuynia cordata* leaf extracts to create highly stable and crystalline AuNPs, which exhibited superior inhibitory effects on Gram-negative bacteria compared to Gram-positive bacteria, resulting in larger inhibition zones [21]. Chen et al. documented the synthesis of CuO NPs utilizing the entire *Houttuynia cordata* plant, showcasing potent cytotoxicity against HeLa cells associated with human cervical cancer [22]. This cytotoxicity was attributed to alterations in the PI3k/AKT/mTOR signaling pathway and the induction of apoptosis. Furthermore, Kim et al. utilized aqueous *Houttuynia cordata* leaf extract to synthesize AgNPs with heightened anti-inflammatory activity [23]. As far as we know, there are currently no published reports on using *Houttuynia cordata* extract for synthesizing microwave-assisted AgNPs and evaluating their potential health benefits. In addition to their biocompatibility against human red blood cells, we report that the HCE-mediated AgNPs, under microwave irradiation, exhibit superior bactericidal activity against various bacterial pathogens, including drug-resistant and non-drug-resistant bacterial strains. The antioxidant performance, fungicidal activity, and potent antibacterial mechanism of as-prepared AgNPs were also evaluated.

## 2. Materials and Methods

### 2.1. Chemicals

All the chemicals used in the experiment were of analytical grade and purchased from Merck. Silver nitrate (AgNO_3_, 99.85%), aluminum chloride (AlCl_3_, 99%), and quercetin hydrate 95% were purchased from Acros Organics, Waltham, MA, USA. The strains of bacteria were obtained from the American Type Culture Collection (ATCC) and cultured according to the guidelines of the Clinical and Laboratory Standards Institute, including ATCC 25922 *Escherichia coli* (*E. coli*), ATCC 27853 *Pseudomonas aeruginosa* (*P. aeruginosa*), and ATCC 25923 *Staphylococcus aureus* (*S. aureus*). Two tested drug-resistant Gram-negative bacterial strains, including colistin- and imipenem-resistant *A. baumannii*, were manually induced until the concentration of antibiotic reached 32 µM at Tzu Chi University, Hualien, respectively. Methicillin-resistant *Staphylococcus aureus* (MRSA107568) was kindly provided from a patient sample at Tzu Chi Hospital.

### 2.2. Preparation of Houttuynia cordata Aqueous Extract

The dried leaves of *Houttuynia cordata* (HC) were purchased from a licensed Chinese herbal medicine store in Shoufeng County. The characterization of this dried botanical material was conducted by the retailer through a visual assessment of its form, shape, and odor. Subsequently, the dried material was finely pulverized into a powder using an electric blender with a power rating of 250 W. To prepare the aqueous *Houttuynia cordata* extracts (HCE), 2 g of dried and powdered HC leaves was decocted in 100 mL of deionized water (DI) and boiled for 30 min at 70 °C. The resulting extracts were cooled to room temperature, filtered through Whatman No. 1 filter paper, and then kept at 4 °C for further use.

### 2.3. Microwave-Assisted Synthesis of HCE-Mediated Silver Nanoparticles

All the syntheses were conducted using the Biotage Initiator+ Microwave Synthesizer (Uppsala, Sweden). The synthesis process was conducted in single-use 10 mL reaction vessels and septa from Biotage, Uppsala, Sweden, which are intended for high-temperature/pressure reactions in the microwave. Typically, 1.87 mL of silver nitrate solution (10 mM), 1.5 mL of HC extract, and 11.63 mL of DI water were added to the reaction tube for the synthesis of microwave-assisted AgNPs using the HC extract (MW HCE-AgNPs). The vessel containing the reagent was subjected to different reaction periods, including 2, 5, 10, and 20 min at 100 °C with a maximum pressure of 15 psi in the Microwave Synthesizer. The HCE-stabilized AgNPs were denoted as MW2 HCE-AgNPs, MW5 HCE-AgNPs, MW10 HCE-AgNPs, and MW20 HCE-AgNPs, respectively. Reflux-method-mediated HCE-AgNPs were used as a control experiment, following the previously reported method [24]. After the completion of the reaction, the colloidal solutions were separated by centrifugation at 10,000× *g* for 20 min. After centrifugation, the obtained HCE-AgNPs were washed two times using DI and stored at room temperature for further characterization and analysis.

### 2.4. Phytochemical Analysis of HCEs 

The as-prepared HC extracts solution (50 mL) was first dried and concentrated using a miVac Duo concentrator. Then, 2 mg of the obtained dried HC extract was suspended in 1 mL of methanol and sonicated for 45 min. After centrifugation at 1000× *g* for 10 min, the supernatant was collected for the following assays. The Folin–Ciocalteu assay was performed to quantify the total phenolic content (TPC) in the HCE according to a previously published protocol [25]. In general, HC extract (0.08 mL), DI water (0.24 mL), Folin–Ciocalteu’s phenol reagent (0.08 mL), and sodium carbonate solution (8%, *w*/*v*, 0.4 mL) were mixed and stirred for 5 min. After adding DI to make the volume of the solution 1.2 mL, samples were incubated for 30 min prior to recording the absorbance at 765 nm. The gallic acid was used as a reference and the TPC value was defined as mg gallic acid equivalents (GAE)/g of dry HC material. The total flavonoid content (TFC) assay was adopted using an AlCl_3_-based colorimetric assay. Briefly, HC extract (0.5 mL) and quercetin standard solution were, respectively, mixed with AlCl_3_ solution (2%, 0.5 mL) and incubated for 60 min. After measuring the absorbance of the solution at 430 nm, the TFC value was defined as mg quercetin equivalents (QE)/g of dry HC material.

### 2.5. Material Characterization

UV-Vis spectra of MW-AgNPs microwave-assisted synthesis HCE-AgNPs were recorded using a SpectraMax iD3 microplate reader (Molecular Devices, San Jose, CA, USA). FT-IR measurements were measured using the KBr reference material through the Spectrum One FT-IR Spectrometer (Perkin Elmer Instruments, Waltham, MA, USA). X-ray diffraction patterns (XRD) of HCE-AgNPs were obtained via Cu Kα radiation (λ = 1.54 Å) using a Bruker D2 phaser diffractometer. The chemical composition of as-prepared AgNPs was determined using the K-Alpha X-ray photoelectron spectrometer (Thermo Scientific, Waltham, MA, USA), where the Al Kα line was the excitation source. Zeta potential measurement was performed using the Nano ZS Zetasizer (Malvern Instruments Ltd., Worcestershire, UK).

### 2.6. Evaluation of the Antioxidant Properties

To further understand the potential antioxidant performance of HC extract and HCE-mediated AgNPs, the 2,2-diphenyl-1-picryl-hydrazyl-hydrate (DPPH) assay and Ferric-reducing antioxidant power (FRAP) assay were performed in this study. The DPPH free radical scavenging assay was performed by mixing HCE or HCE-AgNPs with 0.04% (*w*/*v*) DPPH solution for 30 min at room temperature in the dark. Ascorbic acid was adopted as a reference [26]. The absorbance value for each sample was recorded at 521 nm. The DPPH scavenging activity was estimated as:

DPPH efficiency (%) = [(A_0_ − A_t_)/A_0_] × 100

where A_0_ is the absorbance of the control and A_t_ is the absorbance of the tested sample.

The antioxidant activity of HCE and HCE-AgNPs was examined using the FRAP assay according to previous reports [26]. In general, the tested sample solution (75 µL) was first mixed with a freshly prepared FRAP reagent (650 µL) at 37 °C for 30 min. After incubation, the absorbance value for each sample at 593 nm was determined, and ascorbic acid was used as a standard control.

To determine the Ag ions released from MW-HCE-AgNPs, MW10 HCE-AgNPs at a concentration of 200 ppm were initially dispersed in DI water. Subsequently, aliquots of the MW10 HCE-AgNPs solution (5 mL) were collected at intervals of 0.5 h, 1 h, 3 h, 6 h, and 24 h time periods. Following centrifugation at 15,000× *g* for 20 min, the resulting supernatant solutions were gathered and diluted with 5 M nitric acid prior to performing the atomic absorption spectrophotometry measurement [27].

### 2.7. Evaluation of the Antibacterial and Fungicidal Activity

The antibacterial propensity of HCE and HCE-AgNPs was estimated using the minimum inhibition concentration (MIC) and minimum bactericidal concentration (MBC) tests against all tested bacterial strains. Generally, 1–64 µg/mL of HCE and HCE-AgNPs were incorporated with respective bacterial strains (10^6^ CFU/mL) in the Mueller–Hinton (MH) broth at 37 °C for 18 h. The MIC value was estimated by measuring the optical density (OD) at 600 nm or no visible growth of bacterial strains in three replicate measurements [28]. MBC was determined as the lowest concentration at which no bacterial growth was observed when 200 µL of the bacterial and AgNPs solution, as employed in the MIC assay, was plated on agar. The time dynamic antibacterial test was performed by mixing *E. coli* or *S. aureus* cells (10^7^ CFU/mL) with 4 and 8 μg/mL of MW10 HCE-AgNPs. Untreated corresponding bacterial species were used as a control in the study. After sampling the bacterial solution and indicating the time point, an aliquot of the harvested sample was diluted before culture on the LB plate to estimate the survival rate (%).

The fungicidal efficiency of MW10 HCE-AgNPs against different fungal pathogens, including clinically isolated *Candida albicans* (*C. albicans*) and ATCC 10218 *Trichophyton rubrum* (*T. rubrum*), was evaluated using the colony-forming unit method [29], with slight modifications based on previous literature [30,31]. The fungal pathogens were individually sub-cultured on Sabouraud dextrose agar plates. Fungal colonies were detached from the plates using sterile distilled water before being transferred to sterile tubes. After filtering the fungal hyphae through an 8 µm Whatman nitrocellulose membrane filter, the spore supernatant was collected and then diluted with distilled water to achieve a concentration of 10^5^ CFU/mL. To perform the antifungal assay, 200 µg/mL of MW10 HCE-AgNPs was, respectively, mixed with fungal pathogens (10^5^ CFU/mL), followed by the incubation of the sample at 35 °C (*C. albicans*) and 26 °C (*T. rubrum*) for 18 h. On the other day, 100 µL of each solution was sprayed and incubated on a Sabouraud dextrose agar plate before estimating the survival rate.

### 2.8. ATP Leakage Assay

Bacterial suspensions containing *E. coli* (10^8^ CFU/mL) were mixed with different concentrations of MW10 HCE-AgNPs (0, 4, 8, and 16 µg/mL) in PBS and incubated for 30 min at 37 °C. Following incubation, 200 µL of the solution was mixed with 200 µL of Bactiter–Glo reagent for 5 min at room temperature in the dark. Subsequently, 200 μL of the resulting solutions were transferred into a 96-well plate, and luminescence was measured at 400–750 nm using a SpectraMax iD3 microplate reader [27].

### 2.9. Reactive Oxygen Species (ROS) Measurement

To measure reactive oxygen species, *E. coli* at a concentration of 5 × 10^7^ CFU/mL was mixed with MW10 HCE-AgNPs (80 mg/mL) for 2 h. Following two washes with PBS buffer, the mixtures were treated with DCFH-DA (25 µM) for 30 min. After two additional PBS washes, fluorescence images were captured using an Olympus IX71 microscope (Tokyo, Japan) equipped with a SPOT RT3 digital camera, employing excitation at 510–530 nm.

### 2.10. Electron Microscopy Experiments

MW10 HCE-AgNPs were first subjected to a 30-min incubation at 37 °C with *E. coli* in MH medium. The resulting cell pellets were fixed with a solution containing 2.5% glutaraldehyde in 0.1 M cacodylate buffer and 1% tannic acid for 1 h at 4 °C. Following two washes with DI and PBS, the cells underwent dehydration with ethanol. After critical-point drying and coating with gold, the morphology of bacterial cells was examined using a scanning electron microscope (S-4700; Hitachi, Japan). For transmission electron microscopy, a drop containing the bacteria was deposited onto a carbon-coated grid with 2% uranyl acetate, and the grids were air-dried prior to analysis using a transmission electron microscope (H-7500; Hitachi, Japan) [28].

### 2.11. Hemolysis Assay

The hemolytic activity of MW HCE-AgNPs against human red blood cells (RBCs) was assessed with a modified version of a previously reported method [24]. Briefly, RBCs were first harvested based on the approved protocol (No. IRB105–146A) from the human volunteer, centrifuged for 10 min at 4 °C, at a speed of 3500× *g*, and washed three times with 10 mM of phosphate-buffered saline (PBS) buffer. Subsequently, different concentrations of MW HCE-AgNPs were mixed with 4% RBCs at 37 °C for 1 h. Following the incubation period, the mixture underwent centrifugation at 3500× *g* for 10 min, and 200 μL of the resulting supernatant was transferred to a 96-well plate for measuring the OD6oo value. RBCs treated with PBS (10 mM) and Triton X-100 (0.1% (*v*/*v*)) solution served as a control to determine 0% and 100% lysis efficiency, respectively [28].

## 3. Results

### 3.1. Studying the Phytochemical and Antioxidant Properties of HC Extract

The total phenolic and flavonoid content of the HC extracts was ascertained using the TPC and TFC assays by using gallic acid and quercetin, serving as reference points. Table 1 shows that the phenolic and flavonoid contents of the HC extracts varied, and we found that HCE had a higher phenolic content of 4.80 mg GAE/g than the flavonoid content of 0.81 mg QE/g. In order to obtain a further understanding of the antioxidant potential of HC extract, the Ferric-reducing antioxidant power (FRAP) and 2,2-diphenyl-1-picryl-hydrazyl-hydrate (DPPH) radical scavenging abilities were assessed, as presented in Table 1. The obtained results were compared with ascorbic acid as a control. Compared to using ascorbic acid as a control, the scavenging performance and reducing ability of HCE aqueous extract were estimated as 52.44% and 0.29 ± 0.03, at concentrations of 500 µg/mL and 100 µg/mL, respectively. Our observations are similar to those reported by Nguyen et al. [3].

### 3.2. Characterization of Microwave-Assisted Silver Nanoparticles Using the HCE

**Scheme 1:**
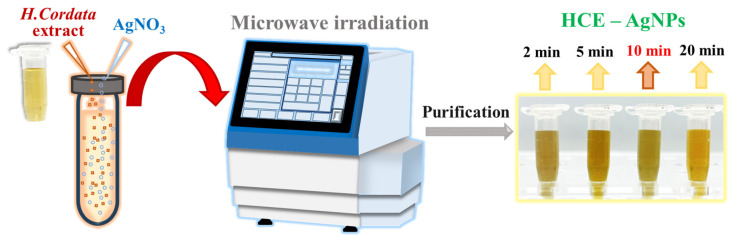
Schematic representation of the fast synthesis of biogenic AgNPs using the aqueous *Houttuynia cordata* extract under a microwave irradiation system.

To further explore the possibility of shortening the reaction time to prepare biogenic AgNPs with enhanced antioxidant performance, we provided a fast synthesis of biogenic AgNPs using an aqueous HC extract coupled with a microwave irradiation technique. In this study, we performed different trials by varying the microwave time from 2, 5, 10, to 20 min at 100 °C. These obtained biogenic AgNPs were named by varying the microwave reaction time, viz., MW2 HCE-AgNPs, MW5 HCE-AgNPs, MW10 HCE-AgNPs, and MW20 HCE-AgNPs. As shown in Scheme 1, with microwave-assisted heating of AgNO_3_ solution and HC extract at 100 °C for 2 min, the colorless solution formed several brownish-yellow colloids, indicating the formation of AgNPs.

**Figure 1:**
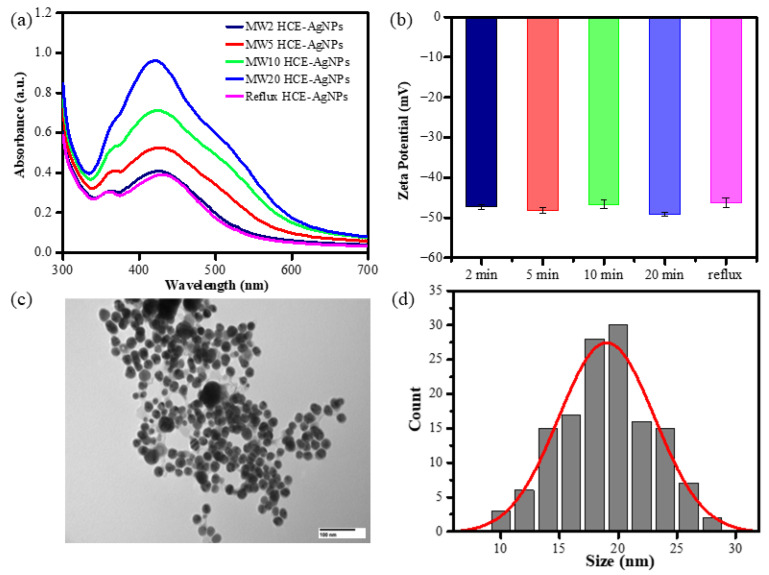
UV-Vis spectra (**a**), Zeta potential measurement of HCE-mediated AgNPs using reflux or microwave-assisted synthesis methods (**b**), TEM (**c**), and size distribution of MW10 HCE-AgNPs (**d**).

Additionally, HCE-mediated AgNP synthesis utilizing a reflux heating system showed that the biogenic AgNPs formed while the solution was incubated at 100 °C for 60 min. As shown in Figure 1, the UV-vis absorption spectra of as-synthesized AgNPs show that the colloids had an obvious absorption band at 430 nm. Figure 1a displays that the SPR peak intensity of microwave synthesis AgNPs increased when the microwave time was extended from 2 to 20 min. It suggests that longer incubation times may promote AgNP formation.

Zeta potential analysis was utilized to assess the comparative surface charge of silver nanoparticles (AgNPs) mediated by HCE in their as-prepared state. The surface potential of AgNPs aided by microwave-assisted synthesis at 2 min, 5 min, 10 min, and 20 min was –47.3 ± 0.5 mV, –48.1 ± 0.7 mV, –46.6 ± 1.0 mV, and –49.1 ± 0.4 mV (*n* = 3), while the AgNPs generated by the reflux method had a surface potential of –46.2 ± 1.2 mV (Figure 1b). To further understand the size and morphology of microwave-assisted biogenic AgNPs, the TEM image of HCE-mediated AgNPs at a 10 min microwave incubation time is shown in Figure 1c. Taking into account around 100 AgNPs, the histogram was created, as shown in Figure 1d, and it unequivocally suggests that the average size dispersion of AgNPs is 19.7 ± 4.2 nm, with a spherical shape.

**Figure 2:**
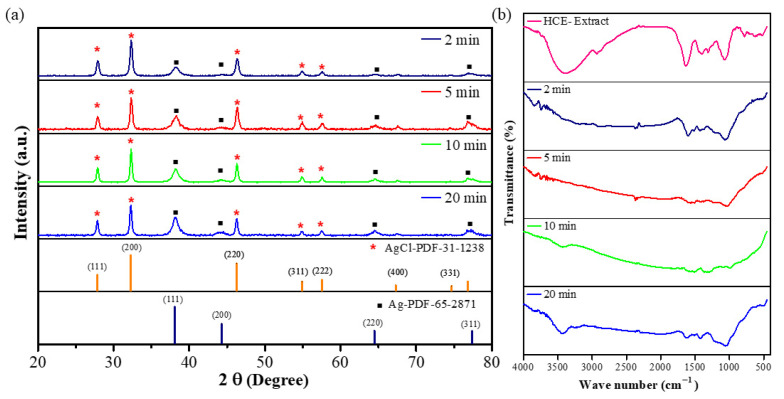
XRD pattern (Red asterisks and black squares indicate AgCl and Ag peaks, respectively). (**a**) and FT-IR measurement for the microwave-assisted synthesis of HCE-mediated AgNPs (**b**).

The crystal phase of as-prepared AgNPs was verified by XRD analysis. The typical XRD pattern of the microwave-assisted AgNPs prepared at different microwave times is shown in Figure 2a. The presence of metallic silver was confirmed by several distinct diffraction peaks at roughly 38.3°, 44.7°, 64.7°, and 77.0°, which are attributed to reflections from the (111), (200), (220), and (311) planes of the silver crystal (PDF-65-2871), respectively [32]. Their indexing as the face-centered-cubic (FCC) structure of silver further supports this identification. The remaining crystalline peaks were at 27.8°, 32.3°, 46.3°, 54.8°, 57.5°, and 67.8°, which were associated with the AgCl planes to the (111), (200), (220), (311), (222), and (400) planes of PDF-31-1238. The XRD pattern reveals that microwave-assisted AgNPs using the HC extract are highly crystalline.

Figure 2b depicts the FT-IR analysis of HCE and microwave-assisted HCE-AgNPs at different microwave periods. The major peaks in the FT-IR spectrum of HCE peaks were observed at 3396 cm^−1^, 2927 cm^−1^, 1630 cm^−1^, 1403 cm^−1^, 1310 cm^−1^, and 1073 cm^−1^. Among them, the functional groups corresponding to 3396 cm^−1^, 2927 cm^−1^, and 1630 cm^−1^ are alcohols (O-H stretching) or amines (N-H stretching), alkanes (C-H stretching), and conjugated dienes (C=C stretching) or amines (N-H stretching). Here, 1403 cm^−1^ corresponds to the polysaccharide-protein of HC extract, and the functional groups corresponding to 1310 cm^−1^ and 1073 cm^−1^ are phenols (O-H bending) and alcohols (C-O stretching), and the corresponding functional groups above are similar to previously reported pieces of literature reported by Kim et al. [23] and Cheng et al. [33]. In addition, we found that microwave-assisted AgNPs had several former peaks from the HCE at the vibration frequencies of 3434 cm^−1^, 1602 cm^−1^, 1370 cm^−1^, and 1000 cm^−1^, respectively, corresponding to alcohols (O-H stretching) and conjugated dienes (C=C stretching) or amines (N-H stretching), polysaccharide proteins of HC extract, and alcohols (C-O stretching). From the shifts and disappearance in the peaks of the HCE-capped AgNPs, it can be presumed that the HCE acts as the reducing and stabilizing agent for the synthesis of AgNPs under microwave irradiation.

**Figure 3:**
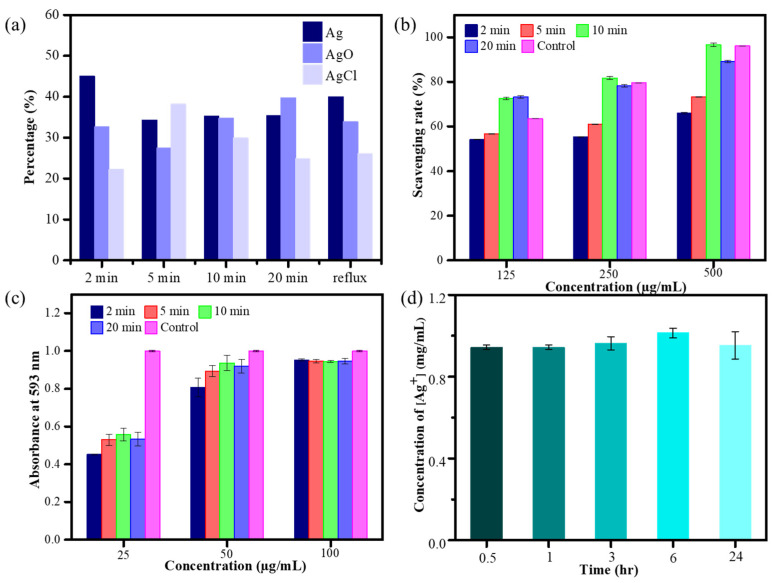
Relative percentage content of Ag components (**a**), DPPH assay (**b**), FRAP test of microwave synthesis of HCE-AgNPs (**c**), and Ag ion release test by MW10 HCE-AgNPs at different incubation times (**d**), with triplicate measurements.

The elemental composition and oxidation state of MW HCE-mediated AgNPs were investigated using X-ray photoelectron spectroscopy (XPS). Figures S1–S5 display the full-scan spectrum of microwave synthesis of biogenic AgNPs and the elemental investigation of carbon (C 1s), chloride (Cl 2p), oxygen (O 1s), and silver (Ag 3d). The XPS analysis of C 1s, Cl 2p, and O 1s signals indicated that C 1s peaks can be deconvoluted into C-H (284.2 eV), C-O (284.8 eV), and C-O-C (286.0 eV). The Cl 2p peaks can be deconvoluted to AgCl (197.7 eV), and O 1s peaks are assignable to, e.g., AgOH (532.2 eV), CHO (532.5 eV), and O-C=O (533.2 eV), respectively. The high-resolution Ag3d spectrum belonging to HCE-mediated AgNPs corresponds to Ag3d_3/2_ and Ag3d_5/2_, with binding energies (BEs) at 373.6 and 367.7 eV. The signals have the potential to be fitted to multiple symmetrical peaks, corresponding to AgO, metallic Ag, and AgCl, with BEs at 367.3, 367.9, and 368.10 eV, respectively. The relative percentage content of Ag, AgCl, and AgO on the surface of as-prepared AgNPs is presented in Figure 3a. Notably, among all microwave synthesis silver nanomaterials, HCE-mediated AgNPs synthesized at 10 min have a similar composition of Ag and AgO on the AgNP surface [34].

### 3.3. Estimation of the Antioxidant Performance of Microwave Synthesis of HCE-AgNPs

To verify whether the antioxidant capabilities of HCE-mediated AgNPs are better than those of the HC extract counterpart, the DPPH and FRAP assays were performed on biogenic AgNPs synthesized using the microwave technique. The outcome of the DPPH assay (Figure 3b) depicts that as AgNP doses increased, the scavenging rate of HCE-mediated AgNPs increased. Additionally, at 125, 250, and 500 μg/mL of biogenic AgNP concentration, the HEC-AgNPs synthesized at a 10-min microwave time offered the best scavenging rates of 72.6%, 81.7%, and 96.5%, which is better than the HC extract (i.e., 52.44%, adding 500 μg/mL of HCE). Moreover, by measuring the reducing potential of antioxidants on reaction with a colorless ferric tripyridyltriazine [Fe3+-TPTZ] complex to produce blue-colored ferrous tripyridyltriazine [Fe^2+^-TPTZ], the ferric-reducing antioxidant power (FRAP) activity of various microwave-assisted AgNPs was determined (Figure 3c). The reducing ability of the tested Ag nanomaterials was correlated with the increase in absorbance at 593 nm [35]. Figure 3c displays that all microwave synthesis AgNPs (125 µg/mL) exhibited superior antioxidant performance than the HC extract, and the highest antioxidant capacity was exhibited during the preparation of MW10 HCE-AgNPs.

### 3.4. Amount of Ag Ions Released from Microwave Synthesis HCE-AgNPs

Based on earlier studies, more silver ions (Ag^+^) will be released from silver nanoparticles with much smaller particle sizes. Furthermore, apart from the nanoparticles themselves, the release of silver ions from the AgNP surfaces may also play a vital role in the antibacterial action mechanism of the Ag nanomaterials. In this experiment, the amount of silver ions released by MW10 HCE-AgNPs was determined by atomic absorption spectrophotometry. The average concentrations of silver ions released from 200 μg/mL of HCE-mediated AgNPs solution were 0.94, 0.94, 0.96, 1.01, and 0.95 μg/mL, respectively, at five time points of 0.5, 1, 3, 6, and 24 h (Figure 3d). This indicates that the biogenic Ag nanomaterials will stably release silver ions in a water-soluble state.

### 3.5. Antibacterial Efficiency of Microwave-Assisted AgNPs Using HC Extract

The antibacterial activity of HC extract and microwave-assisted HCE-AgNPs against various bacteria strains was assessed using minimum inhibition concentration (MIC) and minimum bactericidal concentration (MBC) experiments [36]. An array of different microorganisms, including *Escherichia coli*, *Pseudomonas aeruginosa*, *Acinetobacter baumannii*, *Staphylococcus aureus*, and three drug-resistant bacteria, including colistin- and imipenem-resistant *A. baumannii*, as well as methicillin-resistant *Staphylococcus aureus* (MRSA), were tested. As shown in Table 2, the values of all examined Ag nanomaterials and HC extracts against different bacteria strains for the MIC assay were equal to or less than those for the MBC assay. For example, AgNPs were prepared by HC extract using the reflux strategy, delivering an antibacterial performance of 4–8 µg/mL for all tested bacterial species, whereas MW10 HCE-AgNPs offered superior broad-spectrum antibacterial activity, with MIC values ranging from 2 or 4 µg/mL. Conversely, the MIC value of HC extract exceeded 512 µg/mL against every tested strain of bacteria. Notably, after storage of the MW10 HCE-AgNPs at room temperature for more than 160 days, MW10 HCE-AgNPs retained the antibacterial performance compared to the freshly prepared ones.

**Figure 4:**
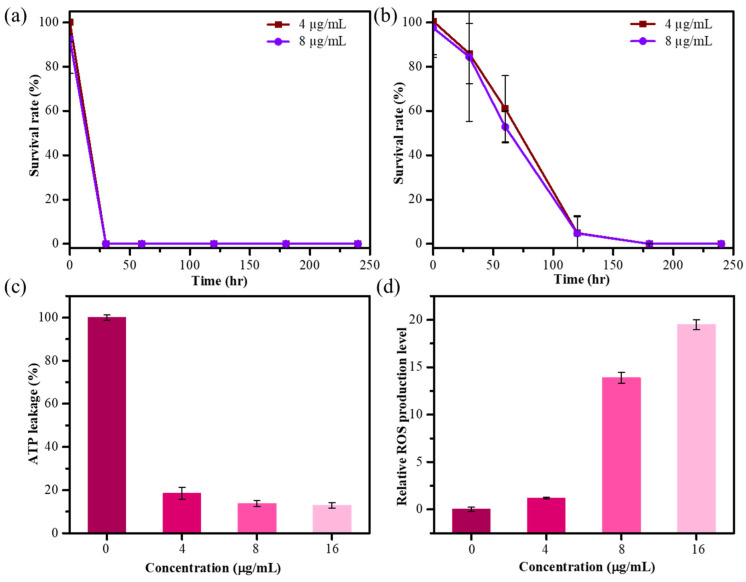
Survival rate curves of *E. coli* (**a**) and *S. aureus* (**b**) after treatment of MW10 HCE-AgNPs with different concentrations (*n* = 3). Results of the ATP leakage assay (**c**) and DCFH-DA test using MW10 HCE-AgNPs treated with *E. coli* (**d**).

The microwave-mediated AgNPs showed superior antibacterial and antioxidant capabilities, so we further examined the time-dynamic antibacterial activity of MW10 HCE-AgNPs against different bacterial strains. Figure 4a demonstrates that the survival rate of *E. coli* dropped to 98% and 92% after being treated with 4 and 8 µg/mL of MW10 HCE-AgNPs for 5 min, while 0% of bacterial cells survived after treatment for 30 min. Figure 4b shows that when *S. aureus* was exposed to Ag nanomaterials, the survival rate decreased to 85% and 84% after 30 min of incubation at 4 and 8 µg/mL concentrations. The survival rate of *S. aureus* was 0% after 3 h of incubation with MW10 HCE-AgNPs.

### 3.6. Evaluation of the Antimicrobial Mechanism of Microwave Synthesis HCE-AgNPs

The release of intracellular components, particularly adenosine triphosphate (ATP), is an essential parameter for determining the degree of bacterial cell death while exposed to nanomaterial. An ATP bioluminescence test was performed to investigate the possibility that cell death was associated with cell membrane disruption. We investigated the effects of varying concentrations of MW10 HCE-AgNPs on intracellular ATP release in *E. coli*. As demonstrated in Figure 4c, the intracellular ATP concentration nearly decreased to 12% after treatment with 16 μg/mL of MW10 HCE-AgNPs. The aforementioned findings revealed that intracellular ATP depletion led to cell death by leaking into the bacterial membrane.

Oxidative stress refers to an imbalance between the production of reactive oxygen species (ROS) and the ability of bacterial cells to damage the bacteria themselves. The 2′,7′ dichlorodihydrofluorescein diacetate (DCFH-DA) assay was used to measure the amount of ROS produced by *E. coli* after treatment with Ag nanomaterials. We investigated the impact of varying the MW10 HCE-AgNPs concentrations on the levels of ROS production in *E. coli* strains. As shown in Figure 4d, an increase in ROS production was observed, indicated by a comparatively higher detected DCF fluorescence intensity with an increase in the AgNP concentration. Evidently, aberrant cell metabolism and function, as well as eventual cell death, are caused by elevated ROS levels.

**Figure 5:**
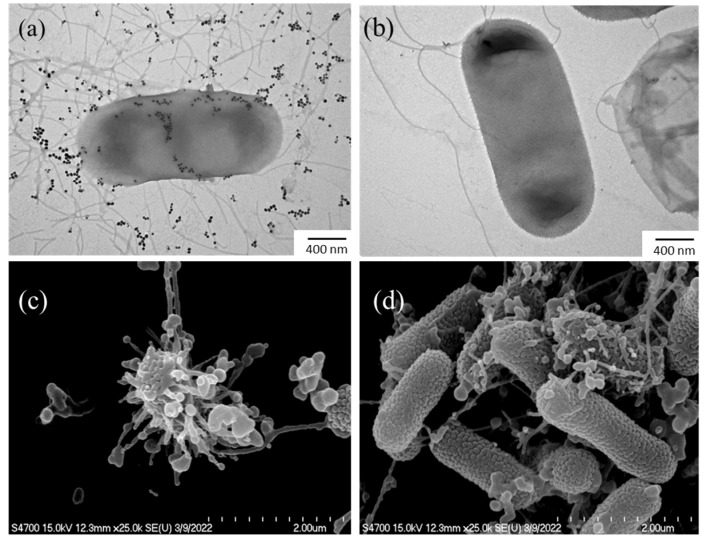
TEM (**a**) and SEM images (**c**) of *E. coli* treated with MW10 HCE-AgNPs, and corresponding TEM (**b**) and SEM (**d**) micrographs of untreated *E. coli*.

Electron microscopy experiments were conducted to investigate the physicochemical changes occurring in bacterial cells during their interaction with HCE-AgNPs. The untreated *E. coli* cells (Figure 5a) were used as a control group for all these morphological changes. As the TEM image is shown in Figure 5b, it was observed that biogenic AgNPs have the potential to adhere to *E. coli* cell surfaces and disrupt the bacterial walls and membranes. Furthermore, SEM measurements (Figure 5c) provided additional evidence that the *E. coli* cells treated with MW10 HCE-AgNPs caused significant structural damage to the bacterial surfaces, resulting in irreversible damage to the cell membrane and the release of cytoplasmic contents [37].

### 3.7. Evaluation of the Antifungal Activity of Microwave-Assisted AgNPs Using HC Extract

**Figure 6:**
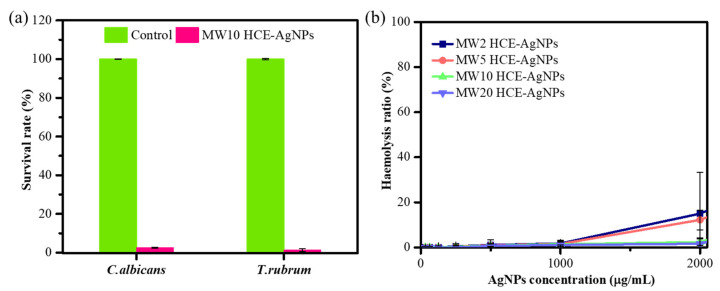
Antifungal activity of MW10 HCE-AgNPs in terms of the survival rate (%) of fungal strains (**a**). Hemolytic activities of microwave synthesis HCE-AgNPs against human RBCs (**b**).

Evaluating the antifungal efficacy of MW10 HCE-AgNPs was also considered crucial. To that end, colony-forming unit (CFU) experiments on agar plates (agar plate method) were conducted against two fungal strains, including *Candida albicans* (*C. albicans*) and *Trichophyton rubrum* (*T. rubrum*) [38]. Compared to the untreated mycological strains, the result in Figure 6a shows that in the treatment with MW10 HCE-AgNPs, the mortality rate was 97.5% for *C. albicans* and 98.6% for *T. rubrum*, respectively. The antifungal efficacy was evident, as the fungal cell viability was nearly negligible at a 200 µg/mL concentration of MW10 HCE-AgNPs.

### 3.8. Evaluation of Hemolytic Efficiency

By culturing human red blood cells (RBCs) with varying concentrations (31.25–2000 µg/mL) of microwave-assisted HCE-stabilized AgNPs, hemolytic evaluations were conducted to ascertain the biocompatibility of the particles (Figure 6b). It is important to highlight that a control group was established by treating red blood cells (RBCs) with 0.1% Triton X-100, aiming to induce complete hemolysis (100%). Figure 6b depicts the viability of the RBCs after treatment with 2000 µg/mL of MW2 HCE-AgNPs, MW5 HCE-AgNPs, MW10 HCE-AgNPs, and MW20 HCE-AgNPs, resulting in 15.03%, 12.22%, 2.39%, and 1.49% hemolysis, respectively. The findings indicate that when the microwave synthesis time increases, the hemolysis rate will progressively decline. As a whole, our findings showed that MW-assisted AgNPs are high-dose, safe antioxidants and antimicrobial nanomaterials.

## 4. Discussion

In an era where drug-resistant bacterial infections are becoming increasingly severe, to prevent the continuous mutation of bacterial genes from producing more drug-resistant strains, it is imperative to find antibacterial agents that can effectively combat bacterial infection. According to our TPC and TFC data, the phytochemical constituents present in aqueous HC extract contain more phenolic compounds than flavonoids. The most important antioxidant components are polyphenolic compounds (including flavonoids) that deactivate free radicals due to their hydrogen-donating ability. A linear relationship between the total phenolic and flavonoid content and antioxidant capacity has been reported in several literature publications [39,40,41].

The majority of phenolic compounds in plants are secondary metabolites and have a significant impact on the antioxidant activity and stimulation of activities associated with the extracts [3]. Additionally, the primary function of the flavonoid metabolites in the plant is to produce yellow pigments. Furthermore, humans can easily consume flavonoids, which are important for their anti-inflammatory, anti-allergic, and anticancer properties [3,9,42]. Nuengchamnong et al. utilized liquid chromatography–mass spectrometry to identify several phytochemicals with antioxidant properties in the aqueous extract of *Houttuynia cordata* [43]. These antioxidants comprised quinic acid derivatives, caffeic acid derivatives, procyanidin B, neo-chlorogenic acid, catechin, chlorogenic acid, crypto-chlorogenic acid, and quercetin hexoside. Additionally, Tian et al. highlighted that the highest antioxidant potential was observed in the water-soluble constituents of HC extract, primarily attributed to acidic polysaccharides [44]. The aqueous HC extract emerges as a source of natural antioxidants, promoting health benefits, and is widely consumed as a beverage in China. Pradhan et al. observed that limonene, bornyl acetate, and methylnonyl ketone within the HC extract exhibited antibacterial effects against Gram-positive bacteria [45]. Although results from antioxidant assays indicate that the as-prepared *Houttuynia cordata* leaf extract possesses substantial antioxidant activity, this plant extract has shown negligible antibacterial activity toward all the tested bacterial strains based on antimicrobial susceptibility testing [46].

Green-synthesized, or biogenic AgNPs, have garnered significant interest due to their effectiveness in treating bacterial infections since natural biological components present in the plant extracts can be employed as reducing and stabilizing agents to prepare silver nanoparticles in a more environmentally friendly manner [12,14,17]. In this study, we found that the reaction time was greatly reduced by using the microwave to generate HCE-AgNPs with enhanced antioxidant and antimicrobial performance compared to the traditional heating method. The thermal capabilities of microwave-assisted synthesis are mainly responsible for the advantages. Microwave synthesis of HCE-AgNPs can benefit significantly from microwave irradiation since the controlled high temperature promotes nucleation, forming more stable nanoparticles. The presence of phytochemicals, such as phenolic compounds, flavonoids, alkaloids, glycosides, terpenoids, amino acid residues, and proteins, are considered to be the factors responsible for reducing silver ions by HC extract [47]. Generally, phenolic compounds are responsible for the reduction of Ag^+^ ions to Ag atoms, which leads to the formation of AgNPs [24].

The UV-visible spectrum showed that microwave-assisted and reflux methods have pronounced absorption peaks at a wavelength of 430 nm, claiming the size and uniformity of silver nanoparticles. The zeta potential value of HCE-AgNPs lies between −46 and −49 mV, which is highly stable in colloidal solution. The numerous negatively charged phytochemicals from HC extract that surround the surface of the AgNPs cause the high negative zeta potential values of AgNPs [48]. Our XPS investigations on microwave synthesis HCE-AgNPs revealed a strong correlation with the XRD findings, indicating the presence of minor AgCl and metallic Ag on the Ag surface of AgNPs. Our findings align with those of Okaiyeto et al. [49] and Devi et al. [50], suggesting that the reaction between Ag^+^ from AgNO_3_ and the Cl– from the phytochemicals in the HC extracts may cause the formation of AgCl. The reducing power of the phytochemicals in the HC extracts allowed the Ag^+^ to be reduced to metallic Ag upon the AgCl formation. Later, Ag^+^ undergoes oxidation and is reduced to Ag atoms, forming an intermediate complex with the phenolic compounds found in the plant extract [49,50].

The DPPH and FRAP assay results displayed that MW10 HCE-AgNPs show high radical scavenging activity and antioxidant efficacy to offer a more significant antioxidant power than other tested antioxidants in a dose-dependent manner. Based on our analysis, the phenolic components present in the HC extract are presumably responsible for the superior antioxidant capacity of MW10 HCE-AgNPs. In addition to donating hydrogen atoms and interrupting the cycle of free radical production to break off chain reactions, phenolic compounds can also prevent oxidative stress [3,47]. The antimicrobial efficacy of Ag nanomaterials is generally highly correlated with their stability in various physiological media. According to our stability test of nanomaterials, MW10 HCE-AgNPs continued to steadily disperse one week after being stored in a range of aqueous solutions.

The MIC results for HC extract and MW HCE-AgNPs against various nondrug-resistant and drug-resistant pathogenic bacteria indicated that the MW10 HCE-AgNPs had higher antibacterial efficacy against all tested bacteria. Alternatively, the synergistic effect of plant extracts and AgNPs amplified plant-extract-mediated AgNPs’ antimicrobial efficacy. Moreover, the influence of silver nanomaterials on bacterial cells and the production of ROS in bacterial cells were also examined. The experimental finding revealed that HCE-AgNPs induce oxidative stress by damaging the cell wall, ultimately resulting in cell death [24,51]. Additionally, ATP, which is involved in the metabolism of cellular energy, was reduced due to the remarkable inhibition by the AgNPs, causing bacterial apoptosis. Electron microscopy findings revealed that MW10 HCE-AgNPs adhered to the outer membrane of *E. coli*, causing the membrane to gradually disintegrate, smooth out, rupture, and release the cytoplasm inside the bacterial cells [52,53].

To test the antifungal properties of microwave synthesis of HCE-AgNPs, *Trichophyton rubrum* ATCC10218 and the clinical strain of *Candida albicans* were used in this experiment. The outcomes met expectations, showing a more than 97% mortality rate. Furthermore, hemolytic studies of microwave-assisted HCE-mediated AgNPs against human red blood cells showed that the HCE-mediated AgNPs were extremely biocompatible with mammalian cells. Thus, phytochemical-capped AgNPs using microwave irradiation have a comprehensive ability to operate as an antioxidant and antimicrobial agent to combat a wide range of bacterial and fungal infections, ultimately defending the environment and community health.

## 5. Conclusions

In the present work, we reported a microwave-assisted strategy to prepare biogenic Ag nanoparticles using aqueous *Houttuynia cordata* extract. Results of phytochemical analyses for HCE revealed that HCE contains more phenolic compounds than flavonoids. Our results further revealed that HCE is an efficient bio-reducing and capping agent in the microwave-assisted synthesis of biogenic HCE-mediated AgNPs. Various spectroscopic techniques were employed for the characterization of the as-prepared HCE-mediated Ag nanoparticles. Microwave-synthesized HCE-AgNPs exhibited exceptional stability and demonstrated superior antioxidant efficacy, potent antibacterial capabilities, and minimal cell toxicity. Our data demonstrated the antimicrobial mechanism of HCE-mediated AgNPs due to the release of active Ag^+^ ions into the bacteria, consequently generating ROS owing to irreversible bacterial membrane damage. Collectively, our results displayed that the aqueous HC extract possessed efficient antioxidant activity for the green synthesis of AgNPs with broad-spectrum antimicrobial capacity and enhanced antioxidant performance.

## Figures and Tables

**Table 1:** Antioxidant properties of HCE.

Sample/Assay	TPC (mg GAE/g)	TFC (mg QE/g)	DPPH (%)	FRAP (Abs. at 593 nm)
HCE	4.80 ± 0.01	0.81 ± 0.01	52.44 ± 0.04	0.29 ± 0.03
Ascorbic acid	N.A.	N.A.	96.09 ± 0.08	1.00 ± 0.03

N.A.: not applicable.

**Table 2:** Minimum inhibition concentration (MIC, µg/mL) and minimum bactericidal concentration (MBC, µg/mL) of HCE-capped AgNPs and HC extract.

Bacterial Species	MW 2 min	MW 5 min	MW 10 min	MW 20 min	Reflux 60 min	HC Extract
Tested assay	MIC/MBC	MIC/MBC	MIC/MBC	MIC/MBC	MIC/MBC	MIC/MBC
*E. coli* ATCC25922	2/2	2/2	2/2	2/2	4/4	>512/>512
*S. aureus* ATCC 25923	4/4	4/4	4/4	4/4	4/8	>512/>512
*P. aeruginosa* ATCC 27853	2/4	2/4	2/2	2/2	4/8	>512/>512
*A. baumannii* ATCC 17978	4/4	4/4	4/4	4/4	8/8	>512/>512
Colistin-resistant *A. baumannii*	2/2	2/2	2/2	4/4	8/8	>512/>512
Imipenem-resistant *A. baumannii*	2/2	2/2	4/4	4/4	8/8	>512/>512
MRSA	4/4	4/4	2/2	2/2	8/8	>512/>512

## Data Availability

Data are contained within the article and Supplementary Materials.

## Supplementary Materials

The following supporting information can be downloaded at: https://www.mdpi.com/article/10.3390/antiox13010032/s1, Figure S1: XPS survey spectra of HCE-AgNPs; Figure S2: XPS C 1s spectra of HCE-AgNPs; Figure S3: XPS Cl 2p spectra of HCE-AgNPs; Figure S4: XPS O 1s spectra of HCE-AgNPs; Figure S5: XPS Ag 3d spectra of HCE-AgNPs.
