# Supplementary material for: Evaluating Antioxidant Performance, Biosafety, and Antimicrobial Efficacy of Houttuynia cordata Extract and Microwave-Assisted Synthesis of Biogenic Silver Nano-Antibiotics
Source: Antioxidants (Basel). 2023 Dec 22;13(1):32. doi: 10.3390/antiox13010032 (PMC10812406; doi:10.3390/antiox13010032)
Supplement: Supplementary file 1 [file antioxidants-13-00032-s001.zip › antioxidants-2772297-supplementary.pdf]

Supporting Information

Evaluating Antioxidant Performance, Biosafety, and Antimicrobial Efficacy of *Houttuynia cordata* Extract and Microwave-Assisted Synthesis of Biogenic Silver Nano-Antibiotics

Kavya Moorthy [1,†], Kai-Chih Chang [2,3,†], Hsiao-Chi Huang [2], Wen-Jui Wu [2] and Cheng-Kang Chiang [1,*]

[1] Department of Chemistry, National Dong Hwa University, Shoufeng 97401, Taiwan; 810712202@gms.ndhu.edu.tw

[2] Department of Laboratory Medicine and Biotechnology, Tzu Chi University, Hualien 97004, Taiwan; kaichih@gms.tcu.edu.tw (K.-C.C.); 109323108@gms.tcu.edu.tw (H.-C.H.); w200811@gms.tcu.edu.tw (W.-J.W.)

[3] Department of Laboratory Medicine, Buddhist Tzu Chi General Hospital, Hualien 97004, Taiwan

* Correspondence: ckchiang@gms.ndhu.edu.tw; Tel.: +886-3-8903622

† These authors share the first authorship.

Table of Contents S2

Figure S1. XPS survey spectra of as-prpeatHCE-AgNPs. S3

Figure S2. XPS C 1s spectra of HCE-AgNPs. S4

Figure S3. XPS Cl 2p spectra of HCE-AgNPs. S5

Figure S4. XPS O 1s spectra of HCE-AgNPs. S6

Figure S5. XPS Ag 3d spectra of HCE-AgNPs. S7

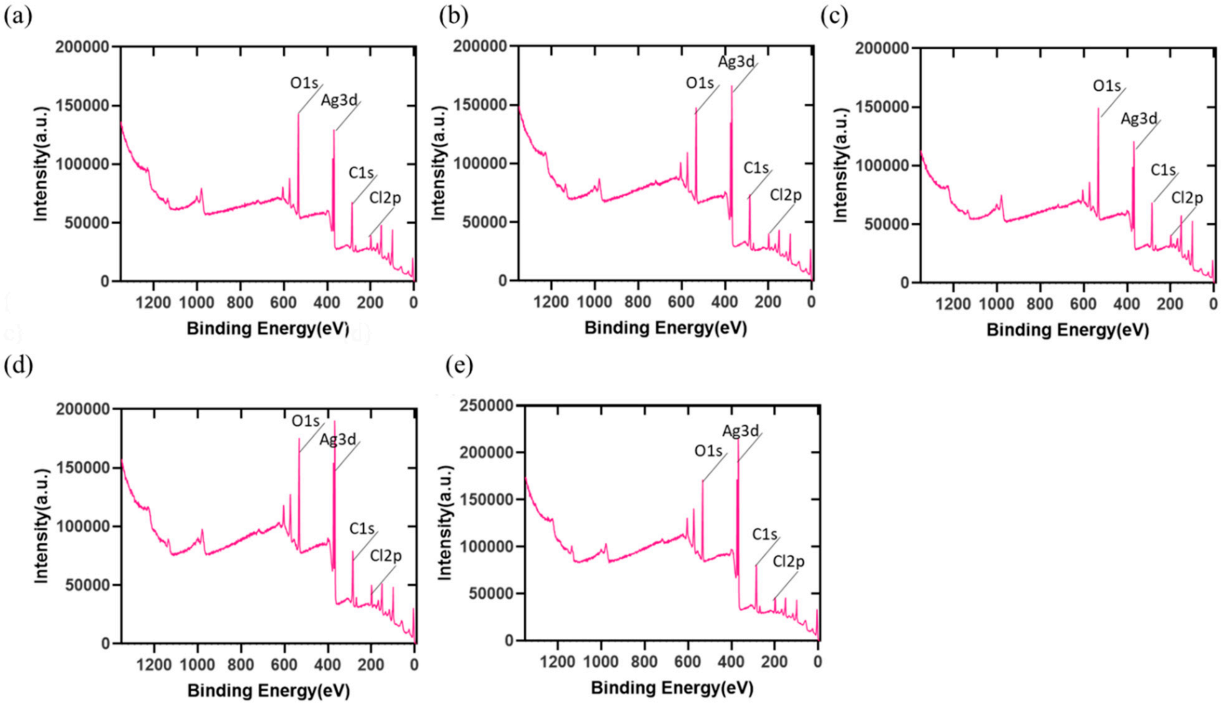

Figure S1. XPS survey spectra of MW2 HCE-AgNPs (a), MW5 HCE-AgNPs (b), MW10 HCE-AgNPs (c), MW20 HCE-AgNPs (d), and reflux method synthesis of HCE-AgNPs (e).

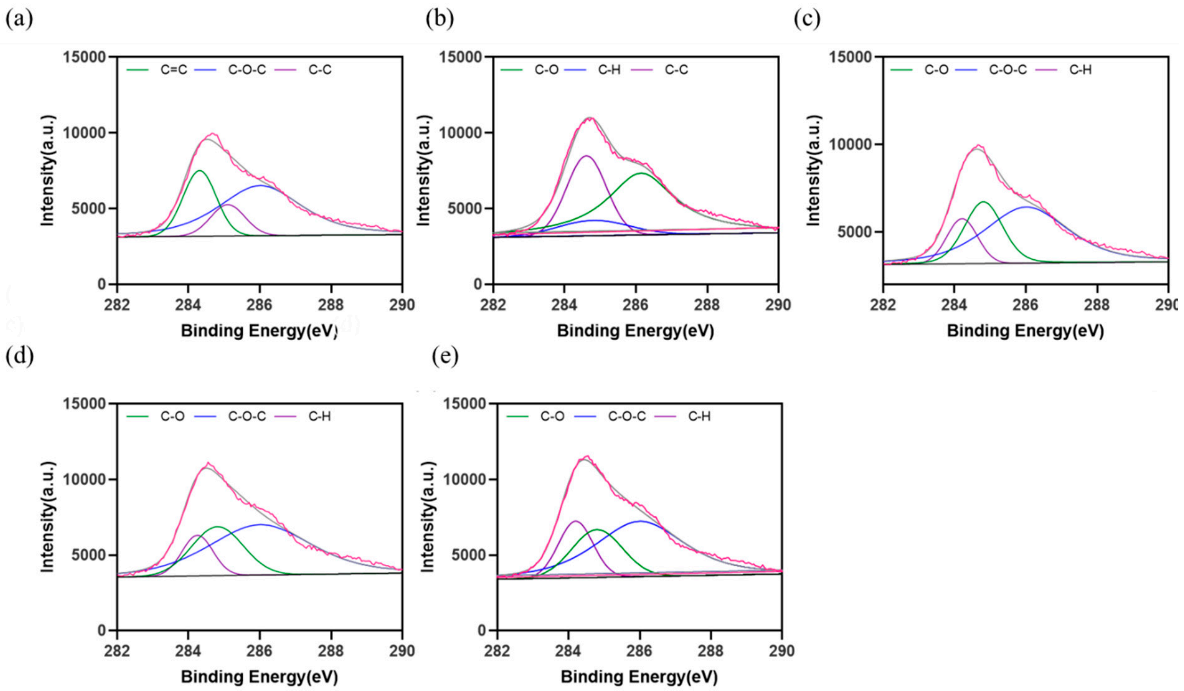

Figure S2. XPS C 1s spectra of MW2 HCE-AgNPs (a), MW5 HCE-AgNPs (b), MW10 HCE-AgNPs (c), MW20 HCE-AgNPs (d), and reflux method synthesis of HCE-AgNPs (e).

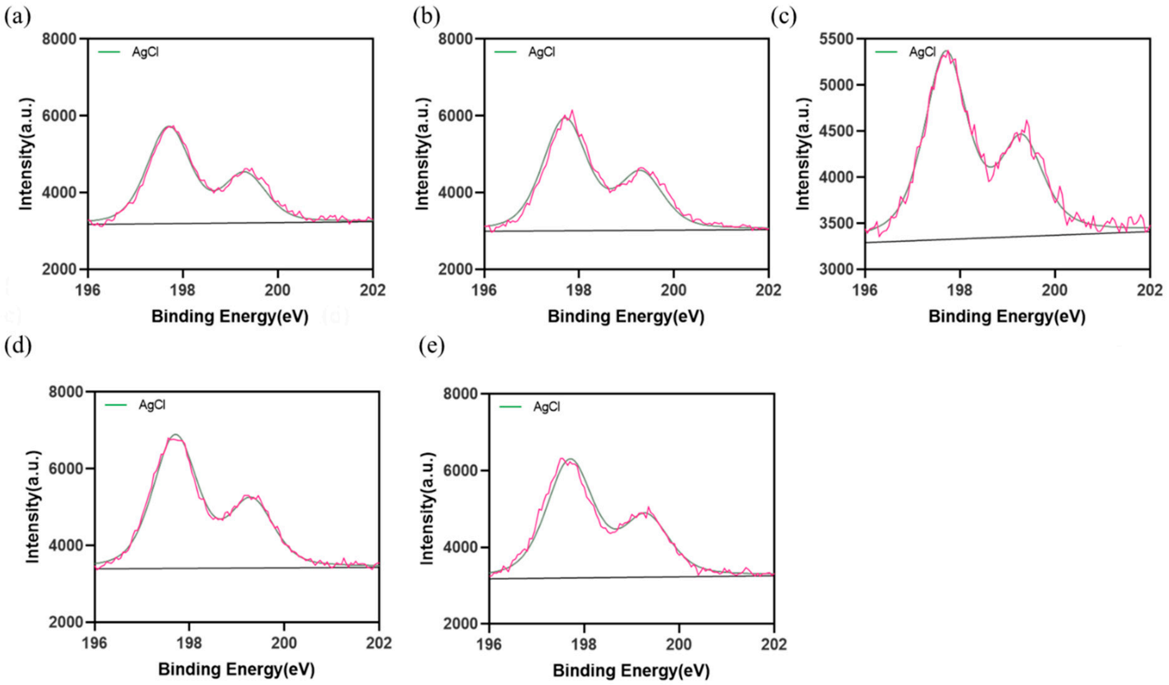

Figure S3. XPS Cl 2p spectra of MW2 HCE-AgNPs (a), MW5 HCE-AgNPs (b), MW10 HCE-AgNPs (c), MW20 HCE-AgNPs (d), and reflux method synthesis of HCE-AgNPs (e).

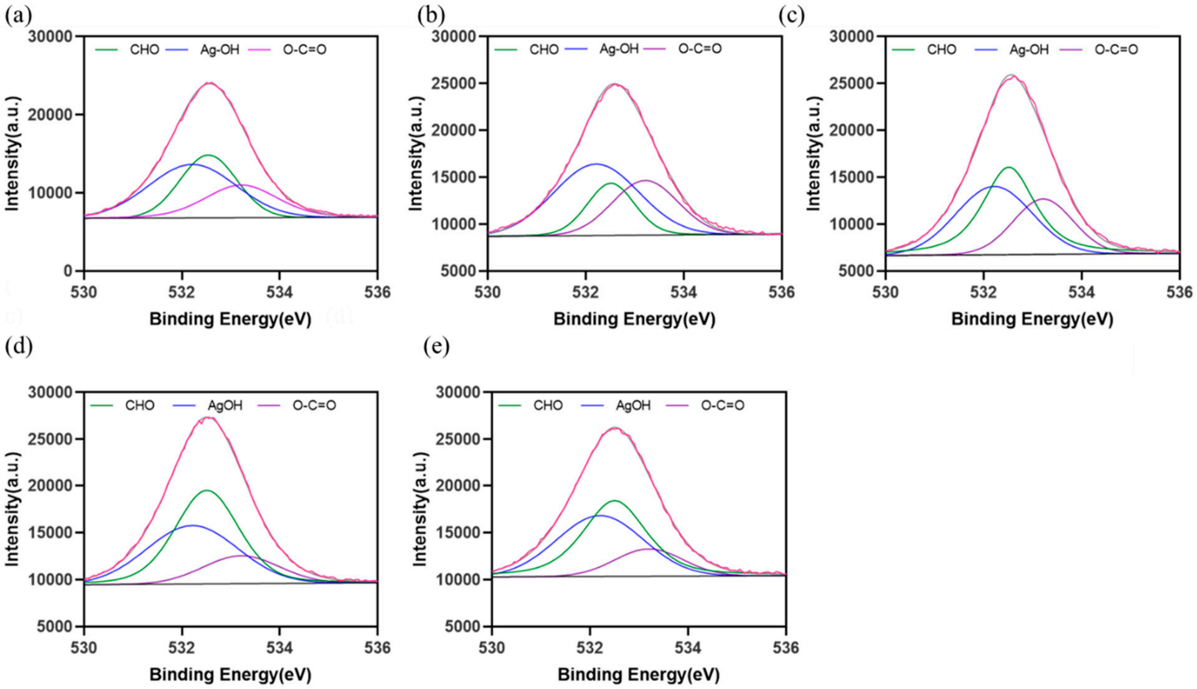

Figure S4. XPS O 1s spectra of MW2 HCE-AgNPs (a), MW5 HCE-AgNPs (b), MW10 HCE-AgNPs (c), MW20 HCE-AgNPs (d), and reflux method synthesis of HCE-AgNPs (e).

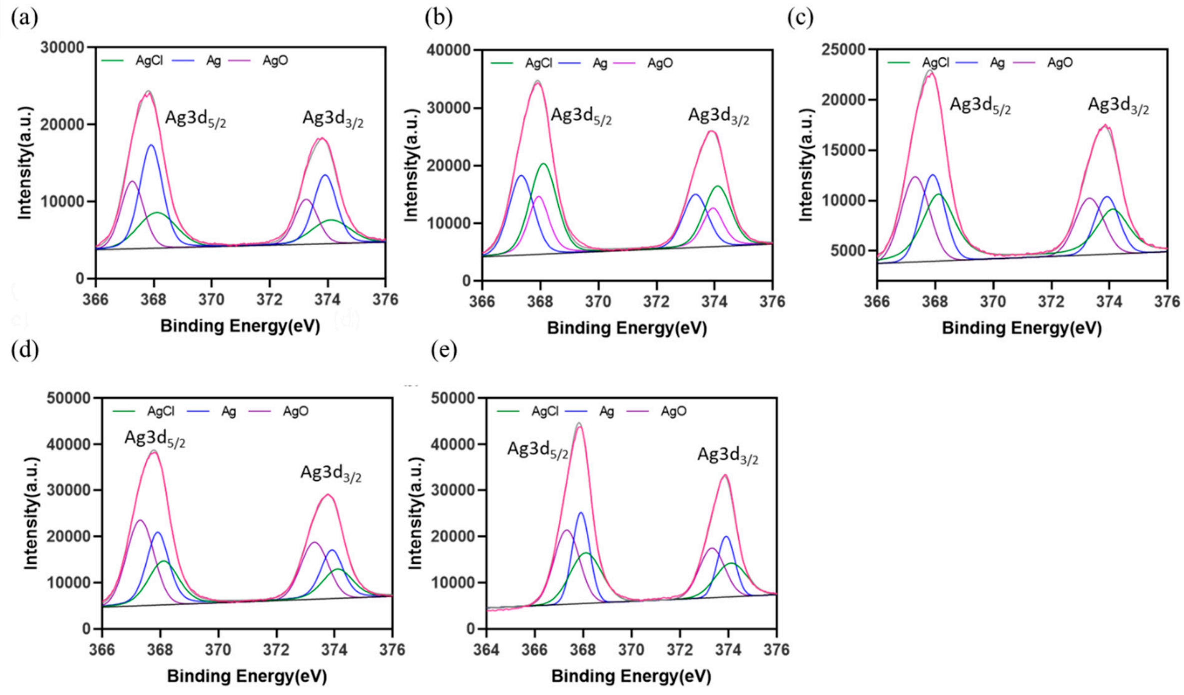

Figure S5. XPS Ag 3d spectra of MW2 HCE-AgNPs (a), MW5 HCE-AgNPs (b), MW10 HCE-AgNPs (c), MW20 HCE-AgNPs (d), and reflux method synthesis of HCE-AgNPs (e).
